# Protective effect of Alpinetin on rats with chronic obstructive pulmonary disease

**DOI:** 10.1002/fsn3.1952

**Published:** 2020-10-25

**Authors:** Yu Su, Xianqi Tao, Jianghui Xu

**Affiliations:** ^1^ Pharmaceutical Administration Section Anqing Municipal Hospital Anqing China

**Keywords:** Alpinetin, apoptosis, COPD, inflammatory factor, pulmonary fibrosis

## Abstract

To investigate the function and mechanism of Alpinetin on chronic obstructive pulmonary disease (COPD) in rat model. The markers of lung injury (body weight/pulmonary function) were measured, and the protein levels of inflammatory‐, apoptosis‐, and fibrotic‐related were determined by Western blot. HE/TUNEL/Masson staining was performed to investigate the mechanisms involved. The levels of inflammatory factors lung injury were detected by ELISA. The in vivo activities of all molecules were determined using a rat model. Alpinetin suppressed the injury of alveolus pulmonis cells occurred in vivo due to the decrease in inflammatory factors and biochemical markers by reduced the expression of TGF‐β1, α‐SMA, and TNF‐α (*p* < .05), associated with the declined of Caspase‐3 and Caspase‐9 (*p* < .01). Additionally, protective affection of Alpinetin downregulated the IL‐6 and upregulated the IL‐10 (*p* < .01). Protective affection of Alpinetin inhibits the apoptosis, inflammation, and fibrosis of alveolus pulmonis cells in rat models.

## Graphical abstract

This work was to evaluate Alpinetin to COPD treatment by vivo study.

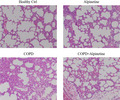

## 1 INTRODUCTION

Chronic obstructive pulmonary disease (COPD) is a typical obstructive pulmonary disease. It is often accompanied by respiratory problems and airflow obstruction, which is mainly manifested as shortness of breath and expectoration (Graf et al., 2018; Hou et al., 2019). Cigarette smoking, air pollution, and dust remain the main causes of COPD (Soriano et al., 2018). COPD is an important public health issue that has become the third leading cause of death in China (Zhou et al., 2016). According to epidemiological data, its prevalence in China was 8.6% in 2015, with about 9.99 million patients suffering from COPD (Wang et al., 2018; Zhong et al., 2007). Western medicine has achieved certain therapeutic effect in COPD treatment, which, however, has certain side effects that bring great pain to patients. Significantly, traditional Chinese medicine has good curative effect and small side effect in the treatment of COPD, which has been increasingly emphasized. Alpinetin is a natural flavonoid compound, which mainly exists in Zingiberaceae, such as turmeric, cardamom, and radix curcumae. As the extract of traditional Chinese medicine, it has become a research hotspot in recent decades. Prior evidence supports that it can inhibit *Helicobacter pylori*, Staphylococcus, and *Escherichia coli*, and suppresses the aggregation of platelets and the release of various inflammatory factors, so as to exert anti‐inflammatory and antioxidant effects (He et al., 2005; Singh et al., 2007). However, it is still unclear whether Alpinetin has a protective effect on COPD and its molecular regulatory mechanism. At present, the main treatment methods of COPD are smoking control, oxygen therapy, bronchodilator, and respiratory stimulant. Despite certain improvement in clinical effect, COPD still has high incidence rate and mortality that have not improved significantly (Chew et al., 2017; Taichman et al., 2014). It is of great significance to explore the pathological mechanism of COPD for finding effective therapeutic targets. Based on the previous research results, the present study was carried out to investigate the therapeutic effect and mechanism of Alpinetin on COPD model in rats, with the expectation to provide new ideas for the treatment of COPD.

## 2 MATERIALS AND METHODS

### 2.1 Materials

#### 2.1.1 Reagents

The following are reagents: Click‐iT^®^ Plus TUNEL assay kit (Art. No.: C1061; Invitrogen); Hematoxylin‐Eosin (HE) Staining Kit (Art. No.: C0105; Beyotime); Masson Stain Kit Masson Staining Kit (Art. No.: 60532ES58; Yeasen Biotechnology (Shanghai) Co., Ltd.); Caspase‐3 (Art. No.: ab13847), Caspase‐9 (Art. No.: ab202068), TGF‐β1 (Art. No.: ab64715), α‐SMA (Art. No.: ab5694), and GAPDH (Art. No.: 1,056) (Abcam); HRP‐labeled goat anti‐mouse secondary antibody (Santa Cruz); IL‐6 (Art. No.: E‐EL‐R0015c), TNF‐α (EELR0019), IL‐10 (E‐EL‐R0016), and ELISA kit (Elabscience); and AniRes2005 animal lung function analytic system (Beijing Belanbo Technology Co., Ltd.)

#### 2.1.2 Instruments

Electrophoresis instrument and semi‐dry membrane transfer instrument (Bio‐Rad); Gel View 6,000 chemiluminescence imaging system (Guangzhou Yunxing Instrument Co., Ltd.); and Multiskan GO Microplate Reader (Thermo).

### 2.2 Methods

Establishment of rat COPD model by cigarette smoking combined with intratracheal instillation of lipopolysaccharide (LPS).

Forty 3‐week‐old SPF grade *SD* rats were purchased from Beijing Vital River Laboratory Animal Technology Co., Ltd. with the license No. of SCXK (Beijing) 2014–0001. The experimental animals were randomly divided into four groups: healthy control group (Healthy Ctrl group), COPD model group (COPD group), Alpinetin alone group (Alpinetin group), and COPD + Alpinetin group. After 1 week of adaptive feeding, the COPD model of rats was induced by cigarette smoking combined with LPS intratracheal instillation. To be specific, rats in each model group were placed in the self‐made plexiglass smoking box with the lid closed; the Huangguoshu cigarette was then inserted into the cigarette lighter box, and then, the micro‐vacuum pump was opened to pump in the smoke. Twenty cigarettes were lighted at a time (1 hr each) for a continuous 3 months of smoking. Rats were intratracheally instilled with LPS (1 mg/kg). Rats in the Healthy Ctrl group were only infused with normal saline without smoking, and the related experiments were carried out after establishing the rat model of COPD. In terms of medication, Alpinetin was given orally for four consecutive weeks (20 mg/kg), and relevant experiments were carried out subsequently.

### 2.3 Basic quality assurance test of rats

After Alpinetin treatment, rats were weighed before sampling. For the detection of pulmonary function in rats, rats were anesthetized with 2% pentobarbital (30 mg/kg) after 12 hr of fasting. Rats were then fixed on their back, followed by the exposure of the skin and muscles of the neck for endotracheal intubation. The pulmonary function of rats was measured by AniRes2005 animal lung function analytic system. The pulmonary function of rats was evaluated by the ratio of forced expiratory volume in one second (FEV1)/forced vital capacity (FVC).

### 2.4 HE staining

The left lung was washed completely with normal saline and fixed with 10% neutral formalin. The sections were dewaxed with xylene for three times, each time for 15, 15, and 10 min, respectively; benzene removal was then performed using gradient alcohol with 100%, 90%, 80%, and 70% alcohol for 10 min, respectively. After that, the sections were washed with distilled water twice (5 min each); stained with hematoxylin for 15 min, washed with distilled water; followed by color separation with 0.5% hydrochloric acid alcohol, washing with distilled water for 15 min; soaking with 70% and 80% alcohol successively for 10 min, re‐staining with eosin for 1 min, 90% alcohol differentiation for 10 s; dehydrating with 95% alcohol twice, 10 min each, and dehydrating with 100% alcohol twice, 15 min each. After that, the sections were cleared by xylene for three times (10, 15, and 15 min each time, respectively). Sections were then observed following mounting with neutral resin.

### 2.5 Detection of inflammatory factors by ELISA

An amount of 1 ml of blood was taken from the heart of the rat, and the supernatant was taken for the experiment after centrifugation in a low‐temperature centrifuge at 4,000 *g* for 8 min. In the step of loading, the blank well and the sample well of the standard to be tested shall be set, respectively. A amount of 50 μl of the standard was added accurately on the coated enzyme‐labeled reaction plate, and 40 μl of sample diluent was added firstly to the sample well to be tested, followed by the addition of 10 μl of the sample to be tested. In the next step of warm incubation, after the plate was sealed with the sealing film, the samples were incubated at 37°C for 30 min. For liquid preparation, the 30 times concentrated washing solution was diluted with distilled water for 30 times and then reserved for further usage. Afterward, in the step of washing, the sealing film was removed carefully, after which the liquid was discarded and the plate was dried, followed by the filling of detergent in each well. It was reacted quietly for 30 s and then discarded for five repeated times. After drying, 50 μl of developer A was added to each well, followed by the addition of 50 μl of developer B to mix gently, and the development was performed at 37°C in dark for 10 min. Then, 50 μl of stop solution was added into each well to terminate the reaction (at this time, the blue color turned to yellow). With the blank well as the control, the absorbance (OD) values of each well were measured at the wavelength of 450 nm. The determination was carried out within 15 min after the addition of the stop solution.

### 2.6 Detection of cell apoptosis by TUNEL assay

After the rats were killed, the tissues were embedded in paraffin, dewaxed in xylene for 5–10 min, replaced with fresh xylene, and then dewaxed for 5–10 min. The next steps were anhydrous ethanol for 5 min. 90% ethanol for 2 min. 70% ethanol for 2 min, and distilled water for 2 min. An amount of 20 μg/ml of DNase‐free proteinase K was added to the sample, followed by reaction with P0106 immunostaining washing solution at 20–37°C for 15–30 min. After three times of PBS washing, an appropriate amount of TUNEL test solution was prepared and added to the sample, followed by incubation at 37°C in dark for 60 min. The number of apoptotic cells in each group was analyzed by ImageJ image analysis software.

### 2.7 Masson staining

The sections were wetted with distilled water for 30–60 s before staining, and then, appropriate amount of hematoxylin nuclear staining solution was added for 60 s of staining, which was then discarded and washed for 30 s. An appropriate amount of fuchsin acid staining solution was added to stain for 30–60 s, discarded, and then washed with cleaning solution for 30 s. An appropriate amount of phosphomolybdic acid solution was added and then discarded after 6–8 m of separation. A proper amount of aniline blue re‐staining solution to stain for 5 min, which was then discarded and washed with anhydrous ethanol. After drying, the sections were sealed and observed under the microscope. The area of fibrosis (%) in tissues of each group was analyzed by ImageJ image analysis software.

### 2.8 Detection of relevant protein expression by Western Blot

The lung tissues were collected from the same part of rats in the four groups. After three times of PBS washing, and the total protein was extracted by lysate with protease inhibitor. The total protein concentration was detected by BCA kit, and the protein was separated by 10% SDS‐PAGE and then transferred to PVDF membrane by semi‐dry membrane transfer instrument. The 5% skimmed milk was used to block the protein at room temperature for 2 hr, and then, the primary antibody was added, sealed, and reacted at 4°C overnight. On the second day, the corresponding second antibody was added and sealed at room temperature for 1 hr, followed by the final step of ECL addition for exposure and development. The relative expression was calculated by gray value statistics after development.

### 2.9 Statistical analysis

All the experimental data were analyzed by SPSS 19.0, and the experimental results were expressed by mean ± *SD*. One‐way analysis of variance (ANOVA) was used to compare the data with homogeneous variance and normal distribution between groups, or rank‐sum test was used for group comparison. *p* < .05 meant that the difference was statistically significant.

## 3 RESULTS

### 3.1 Alpinetin improved body weight and lung function in COPD rats

**FIGURE 1:**
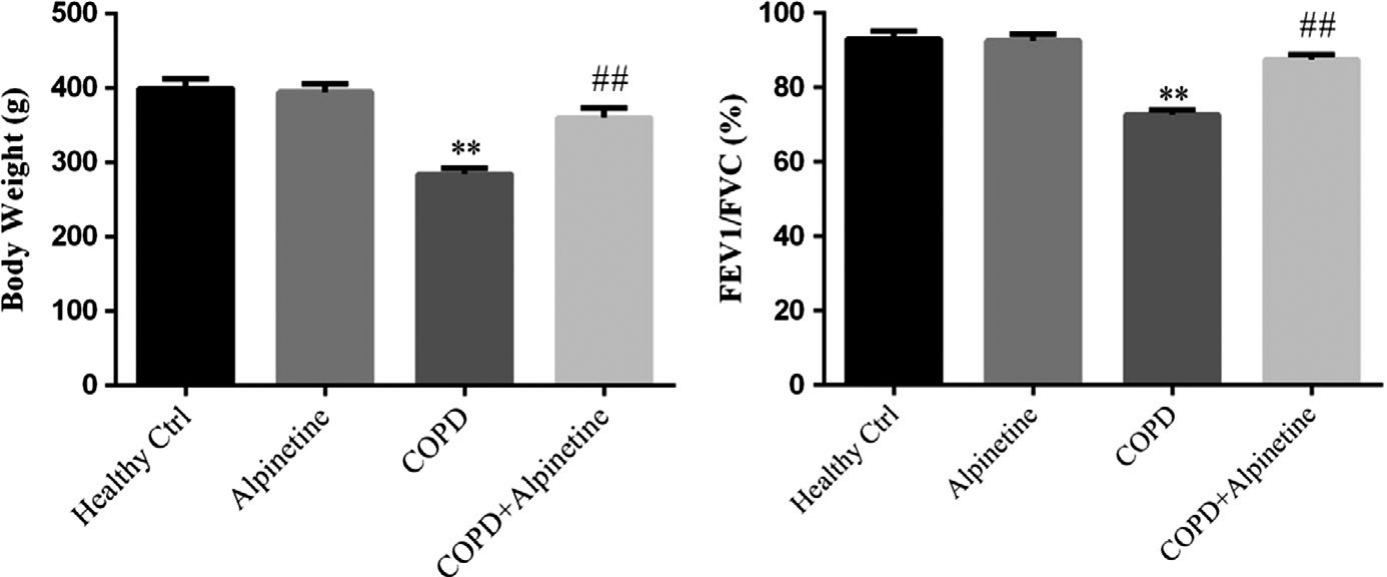
Basic indexes of rats in different treatment groups were measured. Healthy ctrl. Healthy rats; Alpinetin group. Rats received Alpinetin; COPD group. Rats treated by the LPS and cigarettes; COPD + Alpinetin. Rats treated by the LPS, cigarettes and Alpinetin. QT was treated for 4 weeks. ∗∗*p* < .01 compared with the control group; ##*p* < .01 compared with COPD group

There was no difference in the body weight and lung function between Alpinetin group and Healthy Ctrl group after Alpinetin treatment in modeling rats; compared with Healthy Ctrl group, the weight and lung function of COPD group were significantly decreased, while there were obvious improvement in the body weight and lung function in COPD + Alpinetin group when compared with COPD group (*p* < .01, respectively, Figure 1).

### 3.2 Alpinetin improved the pathological changes of lung tissue in COPD rats

**FIGURE 2:**
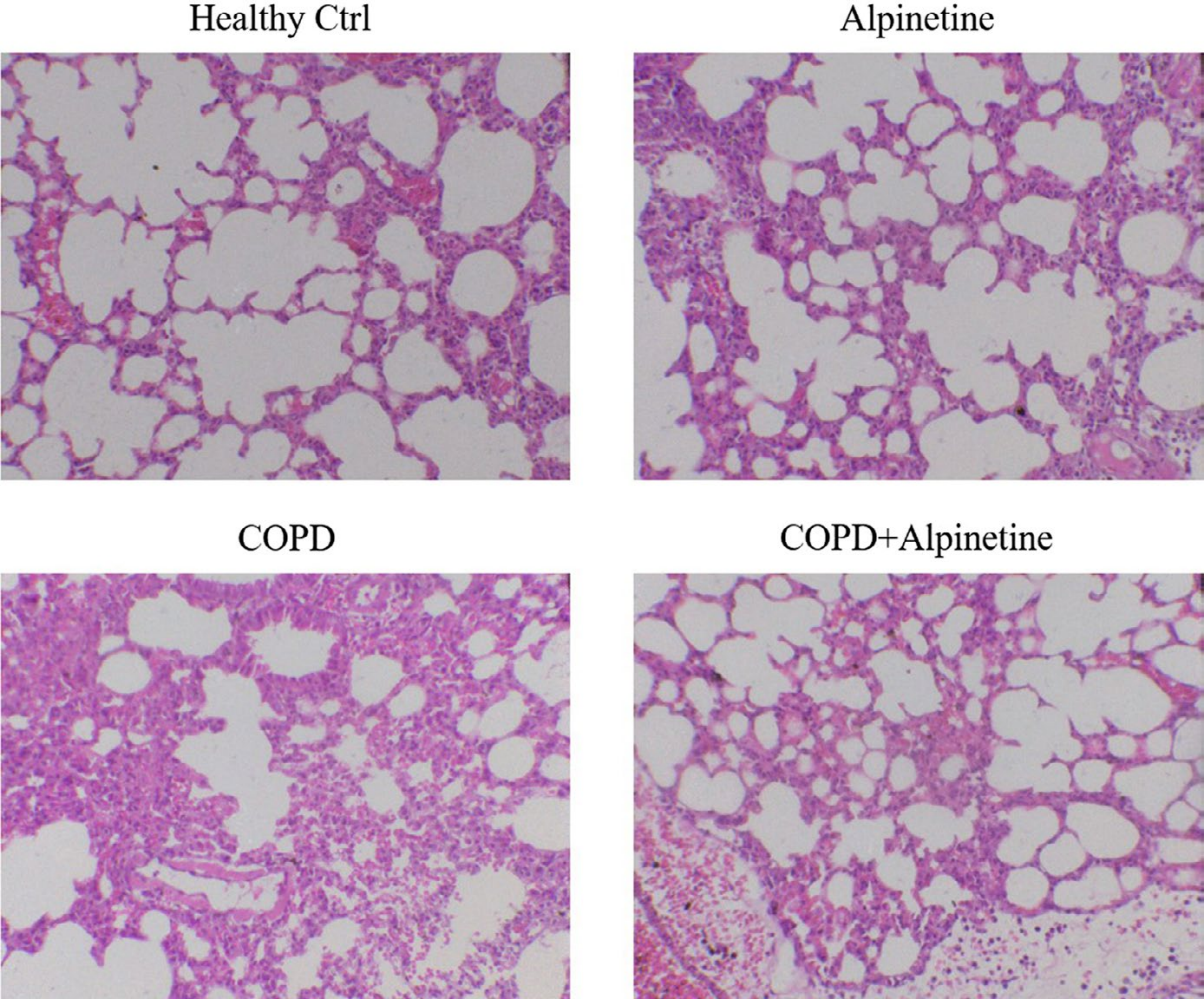
HE staining detected alveolus pulmonis cells morphology (×200). Healthy ctrl. Healthy rats; Alpinetin group. Rats received Alpinetin; COPD group. Rats treated by the LPS and cigarettes; COPD + Alpinetin. Rats treated by the LPS, cigarettes and Alpinetin. QT was treated for 4 weeks

HE staining showed that compared with Healthy Ctrl group, the bronchial lumen, epithelium, and alveoli in Alpinetin group were intact, with no thickening in the wall of the bronchus, and no inflammatory cell infiltration in the submucosa. It suggested that Alpinetin had no obvious effect on the physical signs of rats. Compared with Healthy Ctrl group, the bronchus lumen was seriously deformed, the epithelial cells were obviously exfoliated, the alveoli lumen expanded and fused into pulmonary bullae, the wall of the bronchus was damaged and thickened, and massive inflammatory cells infiltrated around the wall of the bronchus in COPD group. Meanwhile, compared with COPD group, rats in COPD + Alpinetin group showed restored deformation of bronchus lumen, improved exfoliation of epithelial cells, gradually restored and thinned thickness of lumen wall, significantly reduced inflammatory infiltrating cells, and obviously improved expansion and fusion of alveoli lumen (Figure 2).

### 3.3 Alpinetin improved apoptosis of alveolar cells

**FIGURE 3:**
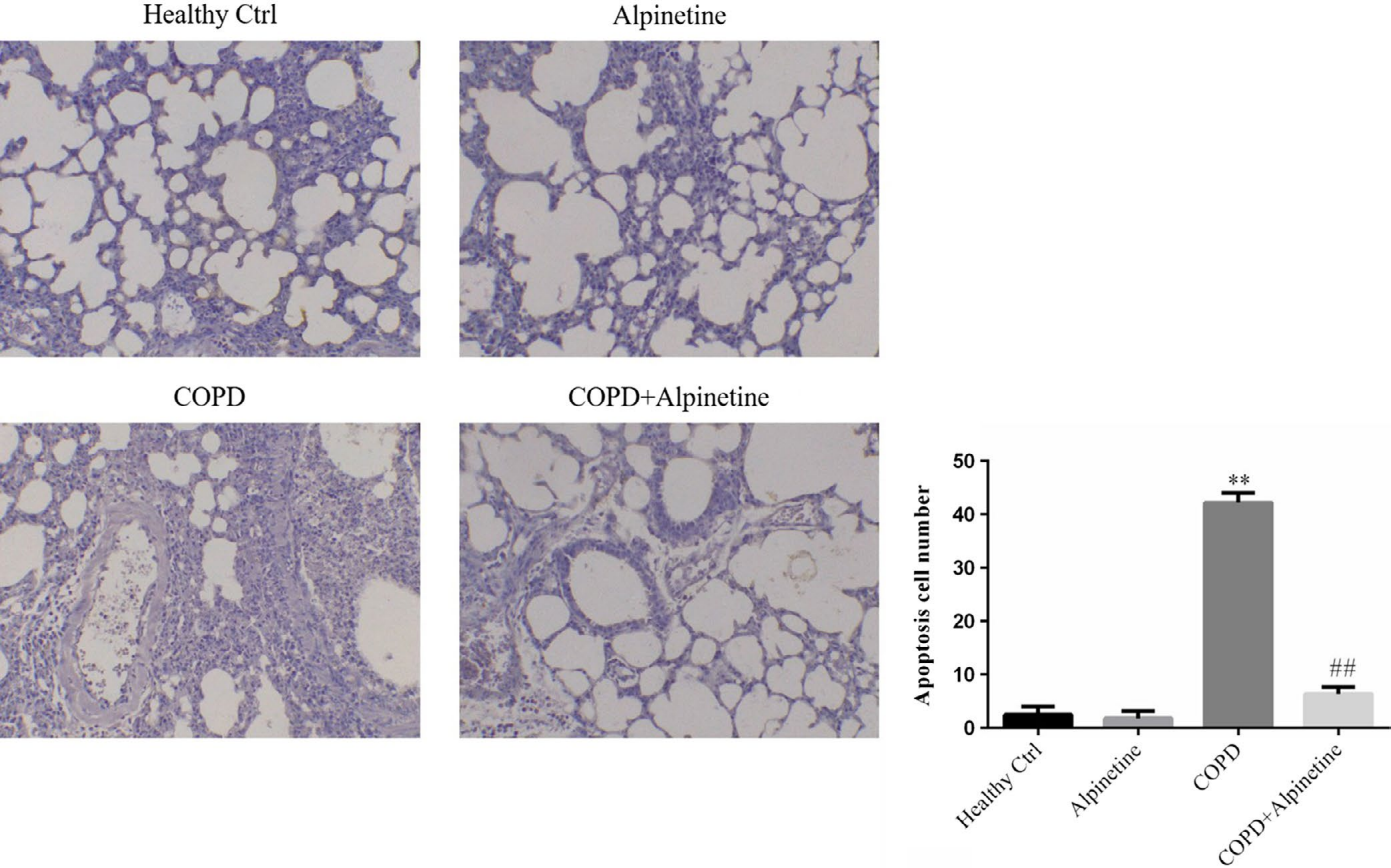
Apoptosis cell number of difference groups by TUNEL assay (×200). Healthy ctrl. Healthy rats; Alpinetin group. Rats received Alpinetin; COPD group. Rats treated by the LPS and cigarettes; COPD + Alpinetin. Rats treated by the LPS, cigarettes and Alpinetin. QT was treated for 4 weeks. ∗∗*p* < .01 compared with the control group; ##*p* < .01 compared with COPD group

UNEL staining showed that there was no obvious difference in the number of apoptotic cells in alveoli between Alpinetin group and Healthy Ctrl group; compared with Healthy Ctrl group, the number of apoptotic cells increased significantly in COPD group (*p* < .01, Figure 3), while there was evident decrease in the number of apoptotic cells in COPD + Alpinetin group when compared with COPD group (*p* < .01, Figure 3).

### 3.4 Alpinetin improved pulmonary fibrosis in alveolar tissue

**FIGURE 4:**
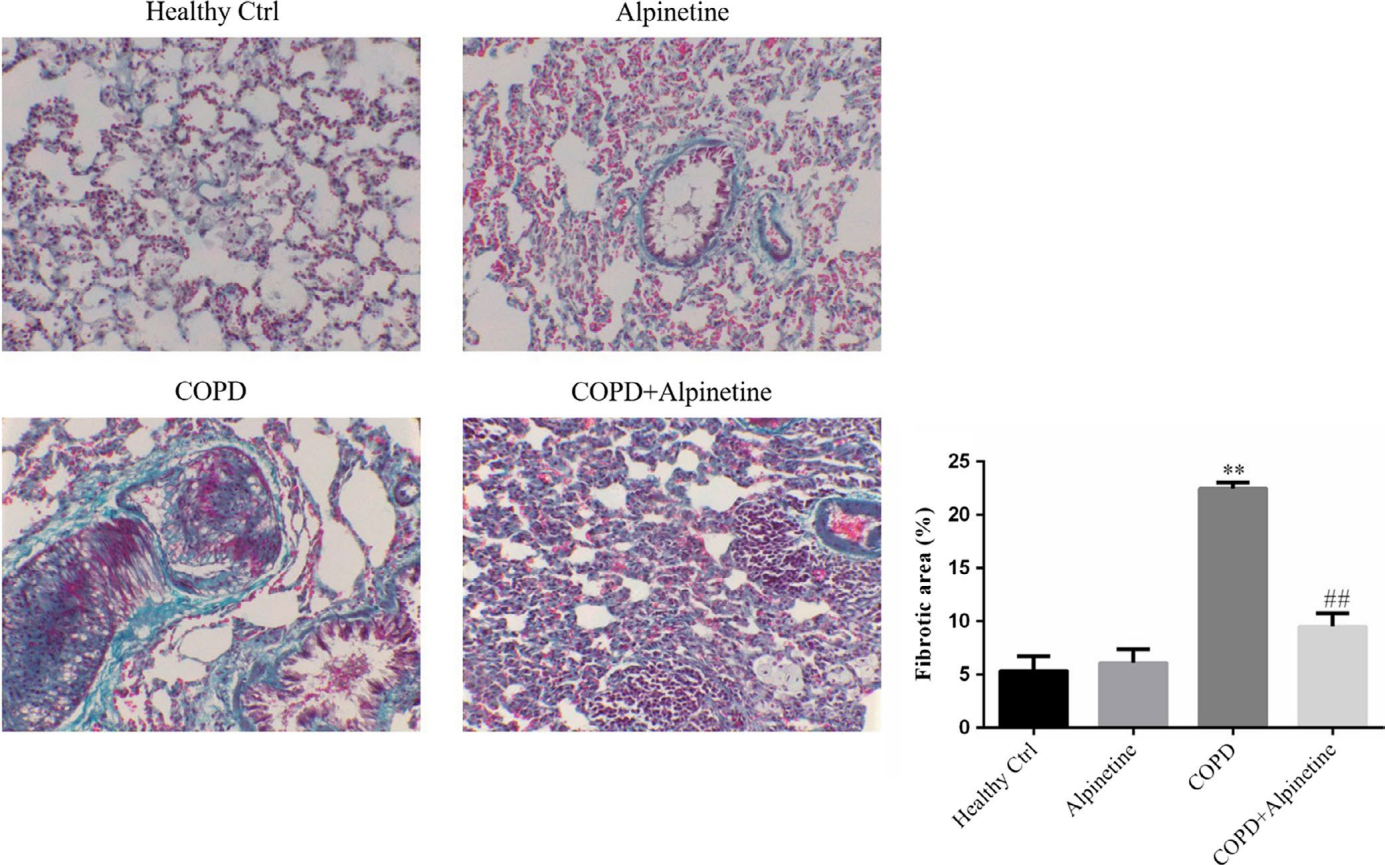
Fibrotic area of difference groups by Masson staining (×200). Healthy ctrl. Healthy rats; Alpinetin group. Rats received Alpinetin; COPD group. Rats treated by the LPS and cigarettes; COPD + Alpinetin. Rats treated by the LPS, cigarettes and Alpinetin. QT was treated for 4 weeks. ∗∗*p* < .01 compared with the control group; ##*p* < .01 compared with COPD group

According to the results of Masson staining, compared with Healthy Ctrl group, alveolar cells in Alpinetin group were rarely stained in blue, and a large number of cytoplasm was stained red; compared with Healthy Ctrl group, there was a larger number of dark blue staining in cells, reduced red cytoplasmic staining, and significantly increased area of fibrosis (*p* < .01, Figure 4). Moreover, compared with COPD group, COPD + Alpinetin group had decreased blue staining of alveolar cells, gradually deepened red cytoplasmic staining, and obviously decreased area of fibrosis (*p* < .01, Figure 4).

### 3.5 Alpinetin improved the expression of inflammatory factors in serum

**FIGURE 5:**
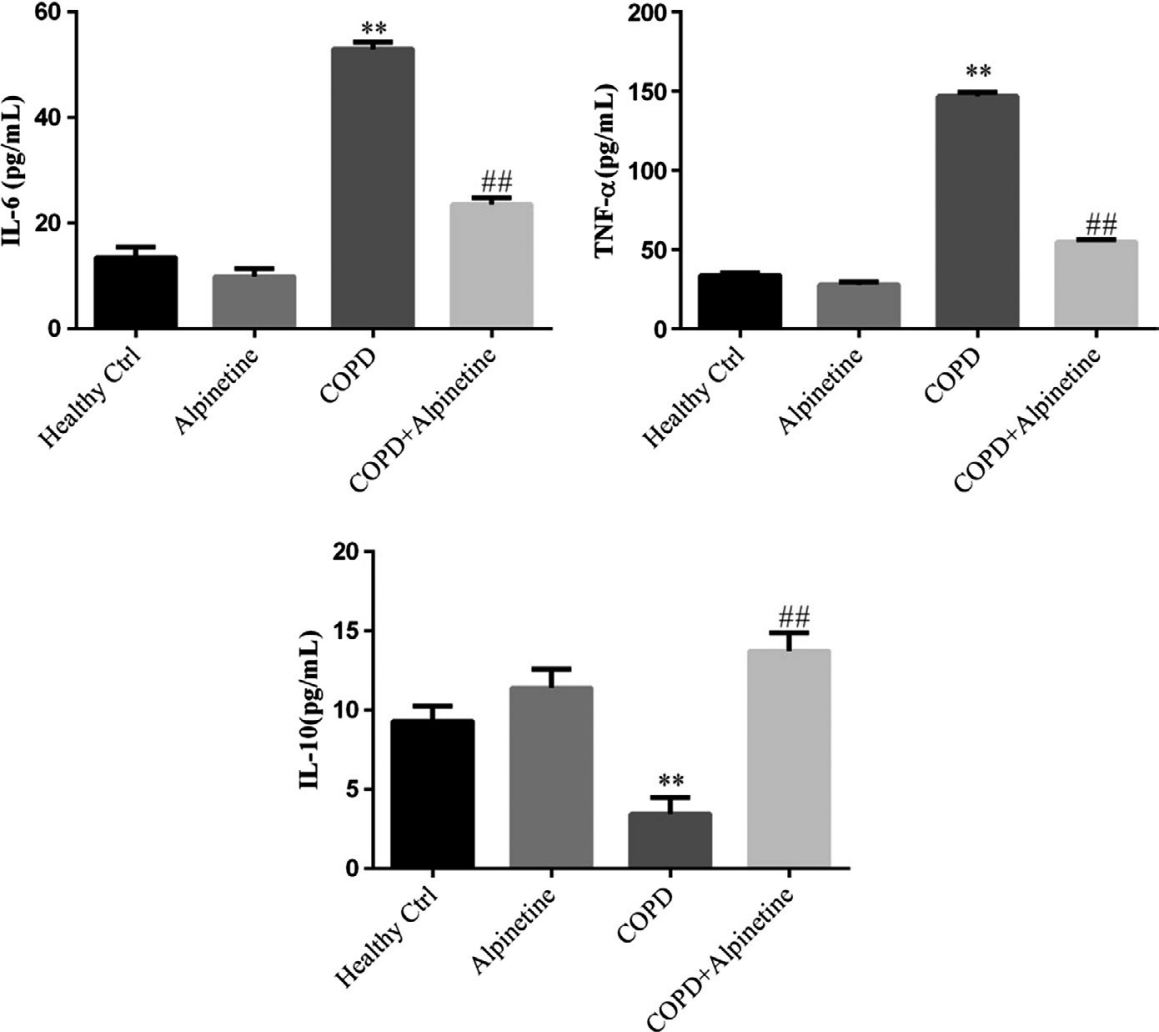
Expression of inflammatory factors were detected in serum by ELISA. Healthy ctrl. Healthy rats; Alpinetin group. Rats received Alpinetin; COPD group. Rats treated by the LPS and cigarettes; COPD + Alpinetin. Rats treated by the LPS, cigarettes, and Alpinetin. QT was treated for 4 weeks. ∗∗*p* < .01 compared with the control group; ##*p* < .01 compared with COPD group

In accordance with the ELISA detection of inflammatory factors IL‐6, TNF‐α, and IL‐10 in the serum, there was no obvious difference in the expression of inflammatory factors in serum between Healthy Ctrl group and Alpinetin group. Furthermore, compared with Healthy Ctrl group, COPD group had significantly upregulated expression levels of pro‐inflammatory factors IL‐6 and TNF‐α, while remarkably downregulated expression level of anti‐inflammatory factor IL‐10. In addition, compared with COPD group, COPD + Alpinetin showed evidently downregulated levels of IL‐6 and TNF‐α, while obviously upregulated level of IL‐10 (*p* < .01, respectively, Figure 5).

### 3.6 Effect of Alpinetin on apoptosis related protein expression

**FIGURE 6:**
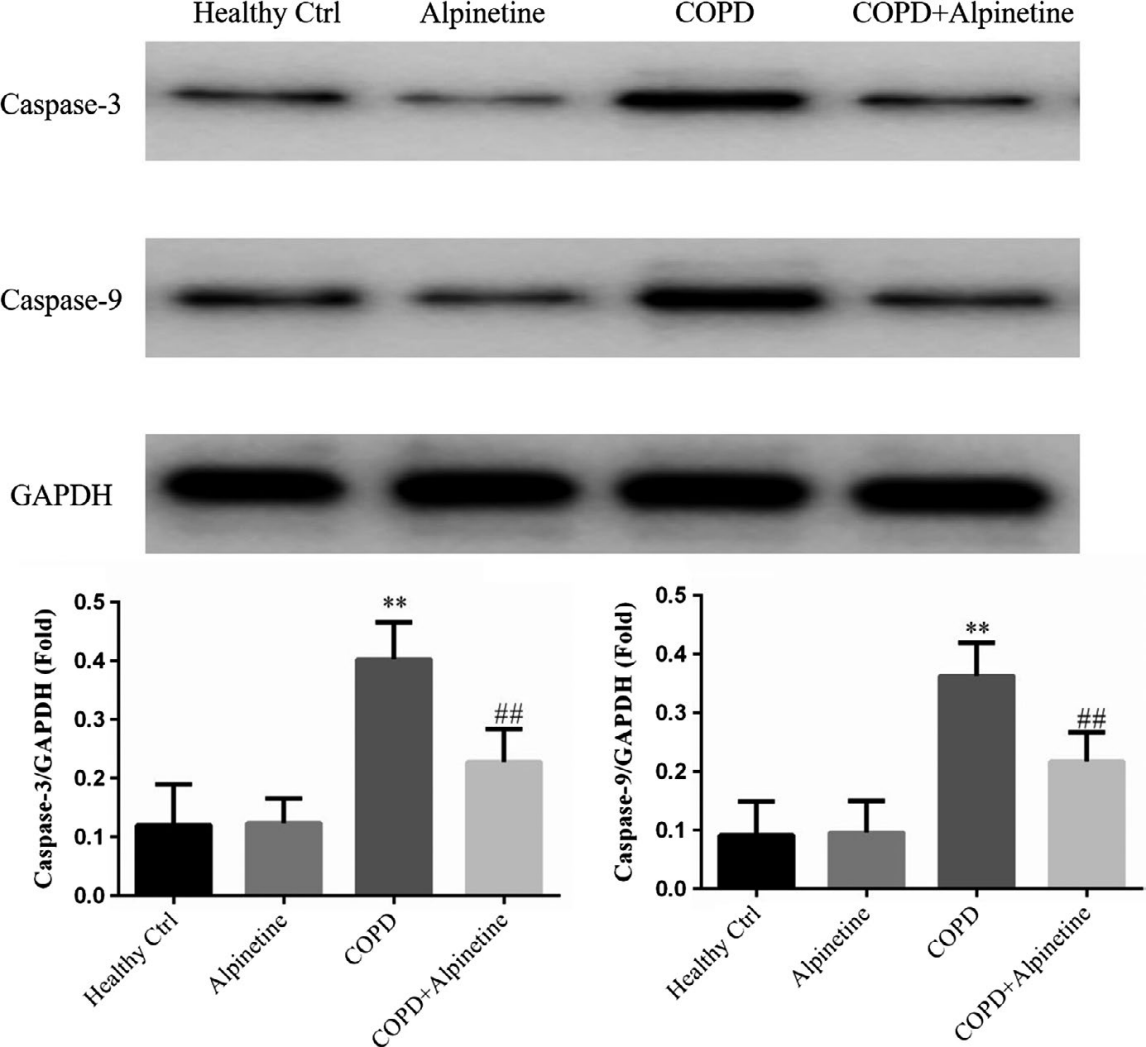
Apoptosis relative proteins expression by WB assay. Healthy ctrl. Healthy rats; Alpinetin group. Rats received Alpinetin; COPD group. Rats treated by the LPS and cigarettes; COPD + Alpinetin. Rats treated by the LPS, cigarettes, and Alpinetin. QT was treated for 4 weeks. ∗∗*p* < .01 compared with the control group; ##*p* < .01 compared with COPD group

Western blot detection results revealed that compared with Healthy Ctrl group, there was no significant difference in the protein expression of pro‐apoptotic factors Caspase‐3 and Caspase‐9 in alveolar tissues of Alpinetin group. Compared with Healthy Ctrl group, the protein expressions of Caspase‐3 and Caspase‐9 were highly increased in COPD group (*p* < .01, respectively, Figure 6). In addition, COPD + Alpinetin group exhibited obviously decreased expression levels of Caspase‐3 and Caspase‐9 than those in COPD group (*p* < .01, respectively, Figure 6).

### 3.7 Effect of Alpinetin on pulmonary fibrosis‐related protein expression

**FIGURE 7:**
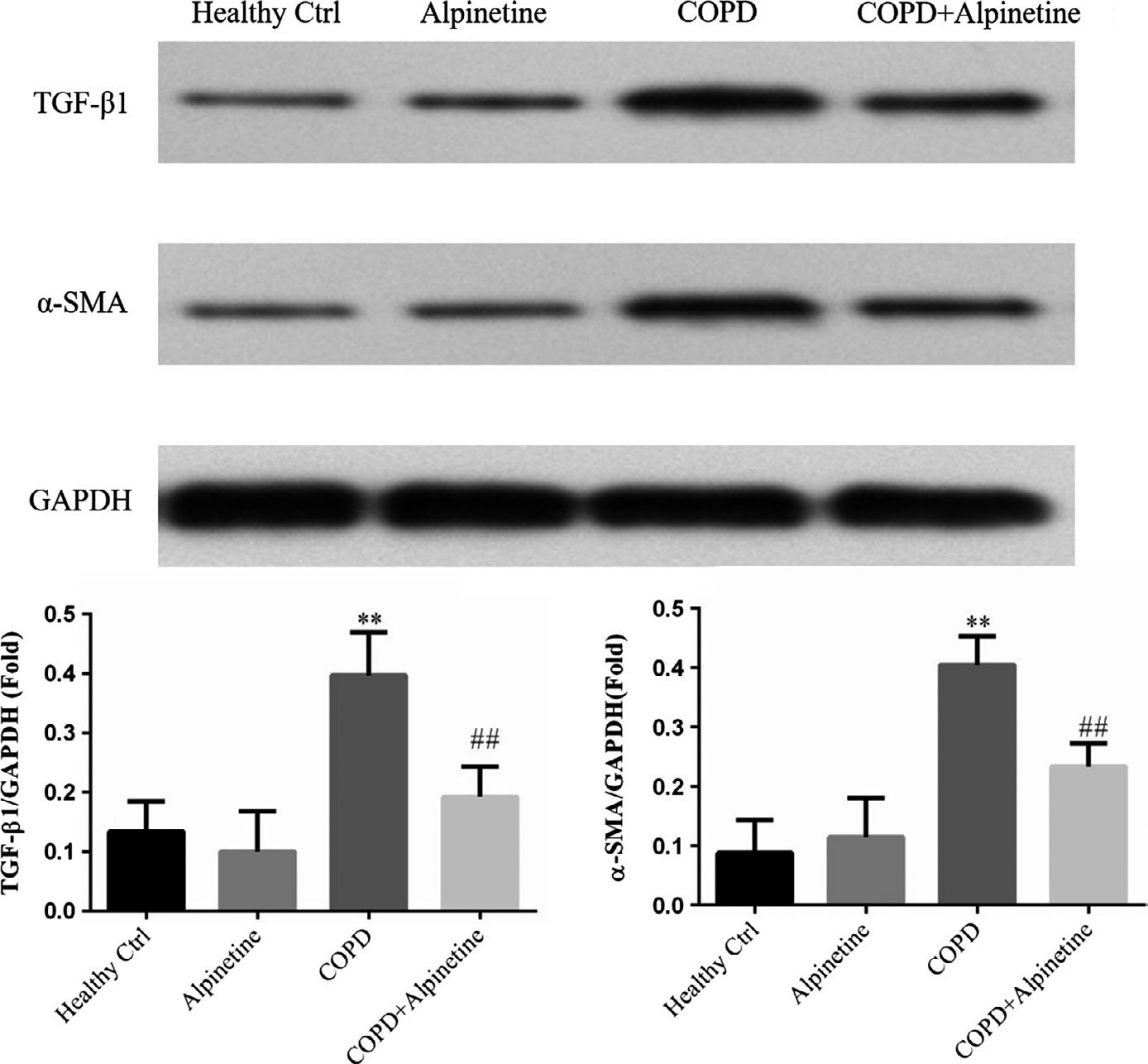
Effect of Alpinetin on pulmonary fibrosis‐related protein expression. Healthy ctrl. Healthy rats; Alpinetin group. Rats received Alpinetin; COPD group. Rats treated by the LPS and cigarettes; COPD + Alpinetin. Rats treated by the LPS, cigarettes and Alpinetin. QT was treated for 4 weeks. ∗∗*p* < .01 compared with the control group; ##*p* < .01 compared with COPD group

According to the results of TGF‐β1 and α‐SMA expression detection in alveolar tissues by Western blot, there was no difference in their expression levels between Healthy Ctrl group and Alpinetin group. Moreover, compared with Healthy Ctrl group, COPD group had significantly increased expression levels of TGF‐β1 and α‐SMA (*p* < .01, respectively, Figure 7). Compared with COPD group, the expression levels of TGF‐β1 and α‐SMA were evidently downregulated in COPD + Alpinetin group (*p* < .01, respectively, Figure 7).

## 4 DISCUSSION

COPD is a progressive disease that eventually evolves into daily attacks, such as dyspnea in patients when walking or with increased mental stress (Mathers & Loncar, 2006), which seriously affects the quality of life in those patients. There are various inflammatory cells in the lung of COPD patients, such as neutrophils, eosinophils, and alveolar macrophages. Activated inflammatory cells by stimulants can produce inflammatory mediators that damage airway walls and lung tissues (Guiedem et al., 2018; Liu et al., 2015). COPD is now recognized as a serious public health problem, which has been listed in the major chronic disease management system by the industry. It has been proved that chronic airway inflammation is the key to the development of COPD, and it progress is related to the abnormal inflammatory response of the lung to harmful particles or gases (Liu et al., 2016; Zheng et al., 2017).

Alpinetin is a kind of natural flavonoid. In recent years, with the in‐depth study of flavonoids, it was found that Alpinetin had antibacterial, antioxidant, anticancer, antithrombotic, hypotensive, hypolipidemic, hypoglycemic, antiemetic, and analgesic effects (Liu et al., 2019; Zhao et al., 2018; Zhao et al., 2018). At the same time, other flavonoids had been found to play a positive role in improving COPD (Bao et al., 2013; Zhao et al., 2019). However, the effect of Alpinetin on COPD is relatively limited. The results of this study had shown that Alpinetin might effectively improve the symptoms of COPD in vivo study.

TGF‐β1 can inhibit cell proliferation and induce epithelial cell apoptosis. Meanwhile, it can induce interstitial‐like cells to proliferate and differentiate into myofibroblasts to promote wound healing and tissue fibrosis (Dong & Ma, 2016). It has been demonstrated that TGF‐β1 was considered to be the most important molecule for promoting fibrogenic growth factor in vivo. For example, there was an increased expression of TGF‐β1 in IPF and bleomycin‐induced pulmonary fibrosis (Khalil et al., 2001). Besides, TGF‐β1 can activate fibroblasts in vitro, and its overexpression in vivo can induce a progressive pulmonary fibrosis (Liu et al., 2001). TGF‐β1 can regulate myofibroblasts by controlling α‐SMA to promote the secretion of extracellular matrix proteins (collagen and Annexin); meanwhile, TGF‐β1 can also regulate or activate the signals of the fibrotic signals activating the function of myofibroblasts (Hinz et al., 2012). TGF‐β1 can reduce the activity of TNF‐α and the inflammatory factor IL‐6 to inhibit the immune inflammation (Bystrom et al., 2018; Jha et al., 2018; Xu et al., 2015). IL‐6 is a multifunctional cytokine produced by many kinds of cells, which can induce the activation of B cells to produce antibodies. It can inhibit extracellular matrix decomposition and stimulate fibroblast proliferation by coagulating collagen, which may lead to airway connective tissue formation and smooth muscle proliferation of COPD, and thus participate in the airway remodeling of COPD (Liu et al., 2016; Zheng et al., 2017). Meanwhile, as a multifunctional anti‐inflammatory cytokine, IL‐10 has important anti‐inflammatory and immunosuppressive effects (Yao et al., 2017). In our study, there was a sharp increase in the expression of TGF‐β1 and α‐SMA. However, both expressions decreased significantly after Alpinetin treatment, which was close to those of normal rats. At the same time, there was an alleviation in the degree of pulmonary fibrosis in experimental rats.

The common apoptotic pathways can be divided into endogenous (mitochondrial pathway) and exogenous apoptotic pathway. Endogenous pathways are often related to cell stress. Under stress, cells may activate BH3, reduce the anti‐apoptotic factor Bcl‐2, so as to reduce the inhibition of Bax, and finally induce Caspase 3/6/7 activation through Caspase‐9 to cause apoptosis. By contrast, the exogenous pathway relies on the binding of FASL, TNF‐α, and TRAIL with death receptor on cell membrane to further activate Caspase‐8 to induce the activation of Caspase‐3/6/7 and thus apoptosis (Teng et al., 2015). In this study, there was obvious downregulation of the expression of Caspase‐3 and Caspase‐9 in COPD rats after Alpinetin intervention. Furthermore, TGF‐β signaling molecule involves in multicellular responses in many important signaling pathways, such as cell proliferation, division, apoptosis, immune, and inflammatory responses (Derynck & Budi, 2019; Ma et al., 2018; Massagué et al., 2005). TGF‐β1 can bind with TNF‐α to inhibit Caspase‐3‐dependent apoptosis pathway to suppress cell apoptosis. In accordance with the results of our study, the expression levels of TNF‐α and TGF‐β1 in COPD rats can be significantly reduced after Alpinetin treatment, and the activity of downstream Caspase‐3 and Caspase‐9 can be effectively reduced, so as to inhibit the occurrence of alveolar cell apoptosis.

In conclusion, Alpinetin has a protective effect on COPD, which can inhibit apoptosis and reduce the release of inflammatory factors by reducing the activities of TGF‐β1, TNF‐α, and α‐SMA. Our study provides reference for the correlation and feasibility of Alpinetin preliminarily in clinical treatment of COPD in the established rat model. However, there is a complex signaling pathway for Alpinetin to function in vivo. It is required to further clarify its related signaling transduction mechanism and screen key targets to provide guidance for precise treatment of COPD.

## 5 STUDIES INVOLVING ANIMAL OR HUMAN SUBJECTS

This study was approved by Ethics committee of Anqing Municipal Hospital, and followed by National Research Council's Guide for the Care and Chinese of Laboratory Animals, the Chinese Public Health Service's Policy on Humane Care and Use of Laboratory Animals, and Guide for the Care and Use of Laboratory Animals.

## CONFLICT OF INTEREST

None.
